# Report from the 1st Cardiovascular Outcome Trial (CVOT) Summit of the Diabetes & Cardiovascular Disease (D&CVD) EASD Study Group

**DOI:** 10.1186/s12933-016-0357-x

**Published:** 2016-02-18

**Authors:** Oliver Schnell, Eberhard Standl, Doina Catrinoiu, Stefano Genovese, Nebojsa Lalic, Jan Skra, Paul Valensi, Antonio Ceriello

**Affiliations:** Forschergruppe Diabetes e.V, Helmholtz Center Munich, Ingolstaedter Landstrasse 1, Neuherberg, 85764 Munich, Germany; Internal Medicine Department, Clinical County Emergency Hospital Constanta, Tomis Blvd. No. 145, 900591 Constanta, Romania; Diabetes and Metabolic Disease Unit, IRCCS MultiMedica, Via Milanese 300, 20099 Sesto San Giovanni, MI Italy; Clinic for Endocrinology, Diabetes and Metabolic Diseases, Clinical Center of Serbia, Faculty of Medicine, University of Belgrade, Dr Subotica 13, Belgrade, 11000 Serbia; 3rd Department of Internal Medicine, 1st Faculty of Medicine, Charles University, U Nemocnice 1, 128 08 Prague 2, Czech Republic; Department of Endocrinology Diabetology Nutrition, Hôpital Jean VERDIER, Paris 13 University, CINFO, CRNH-IdF, Avenue du 14 Juillet, 93140 Bondy, France; Institut d’Investigacions Biomèdiques August Pi I Sunyer—IDIBAPS, Mallorca, 183, 08036 Barcelona, Spain

## Abstract

The 1st Cardiovascular Outcome Trial (CVOT) Summit of the Diabetes & Cardiovascular Disease (D&CVD) EASD Study Group was held during the annual meeting on 30 October 2015 in Munich. This summit was organized in light of recently published and numerous ongoing CVOTs on diabetes, which have emerged in response to the FDA and the EMA Guidelines. The CVOT Summit stands as a novel conference setup, with the aim of serving as a reference meeting for all topics related to CVOTs in diabetes. Members of the steering committee of the D&CVD EASD Study Group constitute the backbone of the summit. It included presentations of key results on DPP-4 inhibitors, GLP-1-Analogues, SGLT-2 inhibitors, acarbose and insulins. Diabetologists’ and cardiologists’ perspective on the potential need of new study designs were also highlighted. Furthermore, panel discussions on the design of CVOTs on diabetes were included in the program. The D&CVD EASD Study Group will continue its activity. In-depth discussions and presentations of new CVOTs like LEADER, will be resumed at the 2nd CVOT on diabetes of the D&CVD EASD Study Group, which will be held from 20–22 October 2016 in Munich (http://www.dcvd.org).

## Background

Historically, the federal drug agency (FDA) Guidance for Industry “Diabetes mellitus: evaluating cardiovascular (CV) risk in new antidiabetic therapies in type 2 diabetes”, issued in 2008 [1], led to the initiation of numerous cardiovascular outcome trial (CVOT) studies on diabetes.

The aim of the Guidance for Industry is to establish the safety levels of newly developed antidiabetic drugs for type 2 diabetes treatment. To that end, sponsors should demonstrate that the therapy will not result in an unacceptable increase in CV risk. According to FDA’s guidance recommendations, to ensure that a new therapy does not increase CV risk to an unacceptable extent, the development program for novel type 2 antidiabetic therapies should include a prospective adjudication of CV events by an independent committee. These events should include CV mortality, myocardial infarction and stroke, but also can include hospitalization for acute coronary syndrome, urgent revascularization procedures, and possibly other endpoints. Patients at higher risk of CV events, such as patients with relatively advanced disease, elderly patients, and patients with some degree of renal impairment are also recommended to be included in the studies.

It is important to notice that the FDA’s guidance documents do not enforce legally binding responsibilities. Rather, it reflects the agency’s standpoint and is to be viewed only as a recommendation.

The Guideline on clinical investigation of medicinal products in the treatment or prevention of diabetes mellitus of the European Medicines Agency (EMA) highlights the need for evaluation of various safety aspects in a dataset representative of this population. It emphasizes that for an assessment of overall safety data in multiple organ systems, it is essential to, as far as possible, exclude that a new drug increases the risk of macrovascular complications, e.g., CV disease [2].

## Main body

The CVOT Summit on Diabetes of the D&CVD EASD Study Group was organized in light of recently published and numerous ongoing CVOTs on diabetes, which have emerged in response to the FDA and the EMA guidelines.

The aims of the CVOT Summit on Diabetes were:

1. Establish a sustainable platform for scientific exchange on CVOTs in diabetes.
2. Support in-depth discussions beyond the level of presentations at large-scale international congresses.
3. Create a network of key stakeholders in the field.
4. Enforce discussions among the scientific community, trial sponsors as well as regulatory and reimbursement authorities.
5. Act as a reference group on matters related to CVOTs on diabetes in the future.

One of the general points of agreement was the importance of differentiating between trials with a primary focus in CVs safety and those that aim to a potential reduction of CV events.

CV safety trials, which assess CV safety of novel drugs, are characterized by a specific study design: They include high-risk diabetic patients and largely aim at glycemic equipoise between active and standard treatment. Therefore studies are designed as non-inferiority comparison trials.

This specific design does not exclude assessment of potential superiority in the trials. Moreover, they also can generate an unprecedented amount of safety data and produce significant data beyond CV outcomes.

The CVOT Summit also discussed trials with a focus on a potential reduction of CV events.

During the 1st CVOT Summit on Diabetes several key questions were debated by diabetologists, cardiologists and initiators of CVOTs:

### Do current CVOTs fulfill the needs of the scientific community?

It was agreed that current CVOTs are truly able to analyze CV safety of novel treatment approaches. Prior to EMPAREG-OUTCOME there was a feeling that given their nature of design FDA-driven CVOTs may be unable to demonstrate superiority in reducing CV risk. As a result, the needs of the scientific community were considered as being largely not fulfilled. EMPAREG-OUTCOME changed this scenario since it showed that empagliflozin treatment produced a reduction of CV events raising the interest of the scientific community for this type of trials.

CVOTs should, by general agreement, address patients’ and physicians’ needs, as well as industry requirements for drug safety evaluation in patient populations, which typify those a physician may encounter in day-to-day practice.

The scientific community may also be interested in studies closer to the real-world setting. Real-world data analyses from adequately characterized data sources, and/or simple pragmatic interventional trials could be helpful in this regard.

The current lack of CVOTs with insulin (except: insulin glargine (ORIGIN), insulin degludec versus insulin glargine (DEVOTE, ongoing)) as well as CVOTs assessing metformin and sulfonylurea was also considered as a drawback for current treatment approaches.

If the aim of current CVOTs is to simply rule out excessive mortality, the general needs of the industry can also be considered to be fulfilled. High investment in CVOTs, however, would only make sense in the future if a study design also enables the potential for additional benefits beyond demonstrating CV safety. However, the high budgetary efforts involved by CVTOs may only be handled by a very limited group of pharmaceutical companies.

### Are the right kind of patients included in current CVOTs?

The fact that currently only high CV diabetic patients are included in CVOTs led to the discussion of whether CVOTs results were translatable to other patient groups.

In case of neutrality of CVOTs results or absence of harm of new treatment approaches, results of current CVOTs could be extrapolated to diabetic patients with a lower CV risk. However, in the event of a CV reduction, these results cannot be translated to patients with a lower CV risk. In this case, separate studies need to be performed in this concrete patient group.

On the topic of drugs affecting primarily glucose levels, which may have an impact on CV outcome after many years, it was mentioned that including a too high risk population may result in a too fast accumulation of events. Subsequently, the interpretation of this data may be less conclusive.

Additionally, the need for a closer look into cancer, pancreatitis, kidney function and microvascular complications was emphasized.

### Is it necessary to modify CVOT design?

The following points and aspects were raised and discussed:

- Primary endpoint of choice should become 3P-MACE consisting of CV death, non-fatal MI and non-fatal stroke.
- Heart failure should not necessarily be included in the first composite endpoint but as a secondary outcome. However, heart failure should be more closely looked into.
- In the future, novel designs may be required in larger populations. Including single-trial evaluation therapeutics of different areas.
- Diverse blood glucose lowering drugs may be compared in a single CVOT. This way a more reliable comparisons among therapeutic approaches will be possible and less patients will be on placebo.
- In order to reduce sample size and patient number requierements, it is recommended the use of innovative designs (e.g., RMET analyses).
- CVOTs should focus more on the standard health care system and real-world settings and not be performed in a rather artificial setting.
- Proposed alternatives to CV safety trials to achieve robust safety data include for real-world data analyses from adequately characterized sources of data, and/or simple pragmatic interventional trials.
- CVOTs and their composite endpoints should be designed according to the mechanism of action of the therapeutic. Outcomes like kidney/renal function, cancer should also be considered in CVOTs, depending on mechanism.
- A need for CVOTs on metformin and sulfonylurea was emphasized.
- Comparison of treatment concepts, e.g., traditional versus innovative approaches, was highlighted.
- Potential of CVOTs for costs reduction was discussed. Another point of the discussion was the need of CVOTs to fulfill the requirements of national reimbursement authorities.

### Will guidelines change based on CVOTs results?

In the future glucose-lowering treatment strategies may also be classified in guidelines based on the presence/absence of CVOTs as well as on the outcome of the respective CVOTs.

In view of EMPAREG-OUTCOME, empagliflozin could be initially recommended by guidelines for high CV risk diabetic patients (secondary prevention). Still the question whether the results reflect a class effect cannot be answered until additional CVOTs with SGLT2-inhibitors are published.

## Conclusion

The 1st Cardiovascular Outcome Trial (CVOT) Summit of the D&CVD EASD Study Group was a highly successful scientific event, which presented key results of recent and ongoing CVOTs in a novel as well as interactive multi-disciplinary setting.

The summit discussed both potentials and limitations of current CVOT study designs. It also produced key implications for future design of CVOTs on diabetes.

The D&CVD EASD Study Group will continue its activity. In-depth discussions and presentations of new CVOTs like LEADER, will be resumed at the 2nd CVOT on diabetes of the D&CVD EASD Study Group, which will be held from 20–22 October 2016 in Munich (http://www.dcvd.org).

## Abbreviations

CV: cardiovascular
CVOT: cardiovascular outcome trial
D&CVD: diabetes and cardiovascular disease
DPP-4: dipeptidyl-peptidase-4
EASD: European Association for the Study of Diabetes
EMA: European Medicine Agency
FDA: Federal Drug Agency
GLP-1: glucagon-like-peptide 1
MI: myocardial infarction
SGLT-2: sodium-glucose cotransporter-2
